# A Series of Questions and Answers for Dental Students

**Published:** 1893-04

**Authors:** 


### Bibliographical.

A SERIES OF QUESTIONS AND ANSWERS FOR DENTAL STUDENTS. Consisting of three parts. By F. J. S. Gorgas, M. D., D. D. S. Published by Snowden & Cowman, Baltimore, and also on sale by the Wilmington Dental Manufacturing Co., Philadelphia.

This is a timely contribution to dental literature. The first book for the Freshman's course is easily digested, but gives a good idea of the foundation work of dental knowledge in anatomy, dentition and a general training. It is a fine preparation for college life. Book second takes up these subjects in a more advanced aspect, adding two or three departments, and Book three in a still more thorough manner treats of dental pathology, oral surgery and practical dentistry. There are few practitioners who might not study all of them with interest and profit. The Freshman's course is $2.00; the Junior's course and the Senior's course are each $2.50.

A good idea of the scope and plan of the work may be had by the following opening page of Part First:

Question. Define anatomy?

Answer. The science relating to the structure of organized bodies.

Q. Define osteology?

A. That part of anatomy which describes the bones.

Q. Number of bones of the head?

A. Twenty-two, eight cranial and fourteen facial bones.

Q. Names of the bones of the head?

A. *Cranial*—1 frontal, 2 parietal, 1 occipital, 2 temporal, 1 sphenoid and 1 ethmoid. *Facial*—2 superior maxilliary, 2 malar, 2 nasal, 1 lachrymal, 2 palate, 2 inferior turbinated, 1 vomer, and 1 inferior maxilliary.

Q. Give the composition of bones?

A. Organic or animal matter and inorganic or mineral matter.

Q. Give the quantity and composition of each?

A. Organic matter about $\frac{1}{3}$, consisting of gelatin fat and vessels. Inorganic matter about $\frac{2}{3}$, consisting of phosphate and carbonate of lime (calcium), fluoride of lime, phosphates of magnesium, sodium and chloride of sodium.

Q. What does a transverse section of bone, examined microscopically show?

A. Haversian canals, canaliculi, lacunæ.

Q. What are Haversian canals?

A. Canals for the passage of vessels.

Q. What are canaliculi?

A. Minute canals connecting the Haversian canals with the lacunæ.

Q. What are lacunæ?

A. Irregular dark spaces arranged circularly around the canals and containing the bone cell.

Q. What is the medulla or marrow of bone?

A. Matter of a yellow color, consisting of fat and extractive matters, occupying the medullary canal, the cancellous texture, and the large Haversian spaces.

Q. What vessels are found in bone?

A. Arteries and veins, and according to some authorities, lymphatics.

Then follows a minute description of these processes and parts.—*Items of Interest.*

---

